# Assessment of miRNA-10b Expression Levels as a Potential Precursor to Metastasis in Localized and Locally Advanced/Metastatic Breast Cancer among Iraqi Patients

**DOI:** 10.1155/2024/2408355

**Published:** 2024-02-28

**Authors:** Mays Abdallah, Ismail H. Aziz, Ahmed Zuhair Alsammarraie

**Affiliations:** ^1^Institute of Genetic Engineering and Biotechnology, University of Baghdad, Baghdad, Iraq; ^2^Oncology Teaching Hospital, Baghdad Medical City, Baghdad, Iraq

## Abstract

Breast cancer (BC) stands as the most prevalent form of carcinoma among women, ranking as the second leading cause of cancer-related mortality in the female population. The objective of this study is to assess the expression of miR-10b and determine its diagnostic and prognostic significance in breast cancer patients across various disease stages. The investigation was carried out in Baghdad at the Oncology Teaching Hospital within Baghdad Medical City and the Oncology Unit at Al-Yarmouk Teaching Hospital. A total of 150 samples were included and divided into two groups: the blood group consisting of 90 samples (including control subjects, localized BC patients, and those with metastatic and locally advanced BC) and the tissue group comprising 60 samples (representing both benign and malignant BC cases). The study spanned from March 2022 to January 2023, with patients' ages ranging from 24 to 75 years. The primary focus of this investigation was to identify the gene expression of miRNA-10b in all sample types. This was achieved by measuring gene expression levels and normalizing them to the level of a housekeeping gene (U6), and quantification was carried out considering the ΔCt value and the fold change method (2^−ΔΔCt^). The results revealed an upregulated fold expression of miRNA-10b, particularly in locally advanced and metastatic BC, where the expression was significantly higher compared to the other groups, with a fold expression of 1.770 ± 0.1070. In localized breast cancer, the fold expression was 1.624 ± 0.064, and in malignant tissue, it measured 1.546 ± 0.06754, all relative to apparently healthy control subjects. In summary, our research provides compelling evidence supporting the classification of miRNA-10b as an oncogenic factor in BC. The central involvement of miRNA-10b in the tumorigenic processes of BC highlights its reference for developing novel targeted therapeutic interventions and detection biomarkers for BC treatment. Notably, elevated expression of miRNA-10b was observed in BC tissues, correlating with an unfavorable distant metastasis-free survival outcome.

## 1. Introduction

Breast cancer (BC) is characterized by the abnormal growth of cells in the breast, leading to the formation of a lump or tumor [1]. The development of breast cancer is impacted by a range of factors, including environmental and genetic factors [2]. Often, BC does not exhibit symptoms when the tumor is small, but a common indicator is the presence of a painless lump. BC can also spread to lymph nodes, resulting in swelling or lumps before the primary breast tumor becomes noticeable. Less common symptoms may encompass breast heaviness or discomfort; alterations in the skin such as swelling, thickening, or redness; and changes in the nipples, including nipple discharge [1].

When cancer cells depart from the ducts or lobules, they have the potential to metastasize via the bloodstream or lymphatic system to different organs, notably the brain, liver, lungs, or bones [3]. Micro- and macroscale metastatic lesions are the end result of a multistage process that begins with local tissue invasion and continues through intravasation into the circulation, extravasation at distant sites, and establishment. Importantly, in organ-specific colonization, cancer cells interact with the surrounding microenvironment [4].

Applying the U6 gene as a standard was a crucial first step in studying miRNA-10b expression; this way, it became much easier to determine the cycle threshold (Ct) value for each gene when the procedure yielded amplification graphs for the target miRNA-10b and U6. Important information on the expression levels of miRNA-10b in the research samples is provided by these data, which help assess the study's results.

Here, on chromosome 2, miR-10b can be found, while miR-10a can be identified on chromosome 17, both microRNAs belonging to the miR-10 family [5]. On chromosome 2q31.1, miRNA-10b is located in the HOXD10 gene cluster, which is sandwiched between the HOXD-4 and HOXD-8 genes [6]. But miRNA-10b tries to silence HOXD10, a gene that has been associated with reducing cell invasion and migration [7]. There is an abundance of miR-10b in the metastatic cancer tissues of many different types of cancer, including colorectal, gastric, pancreatic, and glioblastoma [8]. One of the known functions of miR-10b in cancer is to promote tumor metastasis [9].

The transcription factor TWIST-1 increases levels of miR-10b to downregulate HOXD-10 translation. By influencing genes involved in cell migration and the control of the epithelial-mesenchymal transition (EMT) process, this HOXD-10 suppression is an important mechanism in cancer metastasis. Alpha-3 integrin, matrix metalloproteinases, and RhoC are some of the notable genes impacted by this process [10].

By controlling HOXD10, E-cadherin, and syndecan-1, miRNA-10b significantly increases tumor cell invasion and metastasis in the setting of BC [11, 12]. Invasion and migration are facilitated by miRNA-10b, an important factor in the metastatic phase of breast cancer [13]. miR-10b has been described as an oncogenic miRNA that is overexpressed in different types of cancer and is implicated in tumor invasion and metastasis [14]. In a study by Ma et al., it was found that elevated levels of miR-10b were detected in tumor samples compared to nontumorous adjacent mucosa. More recently, Wang et al. [15] reported higher concentrations of miR-10b in colorectal cancer (CRC) tissues compared to adjacent nontumor tissues and normal tissues from healthy controls.

The purpose of this research was to determine whether or not miR-10b was diagnostically or prognostically significant in 150 breast cancer patients. Blood and tissue samples were collected from individuals between the ages of 24 and 75 between March 2022 and January 2023, representing a range of illness stages. The investigation focused on miRNA-10b gene expression, revealing upregulation, especially in metastatic and locally advanced BC (fold expression of 1.770 ± 0.1070). These findings position miRNA-10b as a potential oncogene in breast cancer, highlighting its role in tumorigenesis for innovative detection biomarker improvement and targeted therapeutic strategies.

## 2. Materials and Methods

This is a case-control study encompassing women across a broad age spectrum, spanning from 20 to 75 years old. Participants for the study were enlisted from both the Oncology Teaching Hospital in Medical City and the Oncology Unit at Al-Yarmouk Teaching Hospital, Baghdad. The study spanned from March 2022 to the conclusion of January 2023, encompassing a total of 150 subjects. These subjects were systematically classified into two principal groups:

(1) Blood group: this cohort comprised 90 samples, encompassing 30 samples from women diagnosed with localized BC, 30 samples from women with locally advanced and metastatic BC, and an additional 30 samples from healthy women serving as the control group
(2) Tissue group: this group included 60 samples, with the following subgroups:
  - 60 samples were obtained from cases of mastectomy for BC, involving the excision of the cancerous mass and biopsies acquired using Tru-cut needles
  - 30 samples constituted the control cases, encompassing instances of fibroadenoma, mastitis, accessory breast, lipoma, benign breast masses, and duct ectasia, as well as cases of quadrectomy and mastectomy due to hemorrhagic cysts and hemorrhagic capsules
  - Normal breast tissues from cases of mammoplasty were collected, and all samples from patients and control groups underwent investigation for miRNA-10b expression using quantitative reverse transcription-polymerase chain reaction (qRT-PCR). For gene expression analysis, we employed the relative quantitation approach, normalizing relative to the expression of a housekeeping gene, specifically U6. Quantification was conducted utilizing the fold change method, denoted as 2^−ΔΔCt^

### 2.1. Sample Collection and Preparation

#### 2.1.1. Venous Blood Samples

An aliquot of 5 ml of venous blood was obtained from each participant, encompassing both control and unhealthy women, utilizing one-use syringes. Subsequently, 0.4 ml (400 *μ*l) of the separated sera was extracted and combined with 600 *μ*l of TRIzol™ Reagent. The resulting lysate was then preserved at -20°C for further analysis.

#### 2.1.2. Collection of Fresh Tissue Samples

Tissue specimens were gathered and introduced into 600 *μ*l of TRIzol™ Reagent. The lysate underwent thorough integration through pipetting maneuvers and was subsequently stored at -20°C and maintained until subsequent examination of miRNA-10b gene expression.

### 2.2. Total RNA Extraction with TRIzol

In this section, we detail the extraction of total RNA, involving microRNAs, from the specimens utilizing the TRIzol™ reagent. The extraction procedure adhered to the manufacturer's protocol provided by Thermo Fisher (USA).

### 2.3. RNA Quantitation by Qubit 4.0

The determination of total RNA concentration was conducted employing Qubit® RNA HS Assay Kits, offering a spectrum of RNA concentrations (low concentration: 4.7-46.1 ng/*μ*l; high concentration). Noteworthy, no discernible discrepancies were identified in the total RNA concentrations between the control and tumor samples. Additionally, the RNA purity exhibited consistent values within the respective groups.

### 2.4. Complementary DNA (cDNA) Synthesis for *miRNA-10b*

The reverse transcription of total RNA into complementary DNA (cDNA) was performed utilizing the TransScript® miRNA First-Strand cDNA synthesis SuperMix kit. This procedure was carried out in a reaction volume of 20 *μ*l, adhering to the manufacturer's instructions. The total RNA volume utilized for reverse transcription was 20 *μ*l, as specified in Table S1. Subsequently, the concentration of cDNA was evaluated for efficiency during the subsequent qRT-PCR steps. It is noteworthy that all steps in this process yielded optimal results, confirming the successful reverse transcription process with efficient cDNA production.

### 2.5. The Primers

The primers used in the study were prepared by Macrogen (South Korea) and maintained in lyophilized form until their utilization. To ensure their specificity, miRNA-10b primers were checked using BLAST (http://blast.ncbi.nlm.nih.gov/BLAST.cgi).

### 2.6. Quantitative Reverse Transcriptase PCR (qRT-PCR)

This technique depends on fluorescent light's measurement to determine the quantity of complementary DNA (cDNA) for a specific gene. The procedure initiated with the extraction of total RNA from the specimens, succeeded by reverse transcription utilizing the High-Capacity cDNA Kit, follows the instructions obtained by the kit. miRNA levels were assessed, and the U6 small nuclear endogenous control gene was amplified to normalize miRNA-10b levels. The qRT-PCR procedure was employed utilizing the smart cycler real-time PCR system from Bioer (Japan). The determination of fold variations and gene expression levels relied on measuring the cycle threshold (Ct), employing reagents from the Wizbio pure™ (SYBR) qPCR Kits. The experimental setup can be elucidated as follows.

Before preparing the qPCR reactions, a thorough mixing of the Wizbio pure (SYBR®) qPCR Master Mix, template DNA, and primers was carried out. The specific volumes for each component were determined as per the values outlined in Table S2. Each reaction was performed in duplicate and included negative control samples, consisting of a nonamplification control (NAC), a nonprimer control (NPC), and a nontemplate control (NTC). Following this, total RNA tolerated reverse transcription employing the TransScript® miRNA First-Strand cDNA Preparation Kit. In the context of quantitative reverse transcription-polymerase chain reaction (qRT-PCR), the assessment of miRNA levels involved the utilization of SYBR Green Reagents.

The next step is the qPCR reaction run; this step entailed running the qPCR reaction with a cycling protocol programmed according to the thermal profile specified in Table 1.

### 2.7. Gene Expression Calculation

For assessing the fold changes in the quantified expression of mature RNAs, we utilized the relative cycle threshold method, denoted as 2^−ΔΔCt^. To assess how gene expression levels changed between samples, researchers used this approach, first detailed by Livak and Schmittgen in 2001 [16]. Below, the details are illustrated.

#### 2.7.1. ΔCt

The Ct value of each gene of interest was subtracted from the chosen normalization factor to obtain the ΔCt, which is also called the “normalized raw data” for each gene.

(1) $$\begin{array}{c} \Delta \text{Ct}=\text{Ct}\left(\text{gene of interest}\right)-\text{Ct}\left(\frac{\text{housekeeping}}{\text{reference gene}}\right). \end{array}$$

The expression ratio was calculated according to the formula:

(2) $$\begin{array}{c} 2^{-\Delta \text{Ct}}=\text{normalized expression ratio}. \end{array}$$

#### 2.7.2. ΔΔCt

Additionally, the 2^−ΔΔCt^ method was employed to compare transcript levels between different samples. The ΔΔCt value was calculated by subtracting the ΔCt value of each test group from the control group:

(3) $$\begin{array}{c} \Delta \Delta \text{Ct}=\Delta \text{Ct}\left(\text{patient}\right)-\Delta \text{Ct}\left(\text{control}\right). \end{array}$$

The fold change value was then calculated from the following equation:

(4) $$\begin{array}{c} \text{Fold change}=2^{-\Delta \Delta \text{Ct}}\text{normalized expression ratio}. \end{array}$$

The direction of the fold change was used to determine if the target gene was upregulated or downregulated. In this case, upregulation of the target gene was indicated by a positive result and downregulation by a negative result. Because 1 was believed to represent the typical value in the control sample, the findings were presented as fold change relative to that value.

### 2.8. Statistical Analysis

By employing the statistical analysis and graphing program GraphPad Prism version 9, the study's data was summarized, analyzed, and displayed. This program was used to help analyze and visualize the experimental data, which led to a strong statistical analysis of the study's findings.

## 3. Result and Discussion

Participants' ages ranged from twenty-four to seventy-five years old, with the females broken down by blood type. The particular BC subgroup's age domain, which ranged from 24 to 75 years, was in line with the total cohort. In contrast, the group of persons with metastatic and locally advanced breast cancer consisted of people aged 25 to 75 years, whereas the control group of healthy women had an age range of 20 to 67 years.

The details of sample distribution based on hormone receptors are found in our previous publication [17].

### 3.1. Expression of *miRNA-10b*

**Figure 1:**
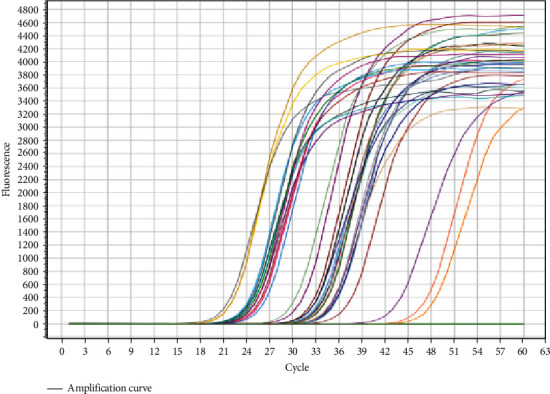
Amplification plots for *miR-10b* expression obtained by real-time PCR.

**Figure 2:**
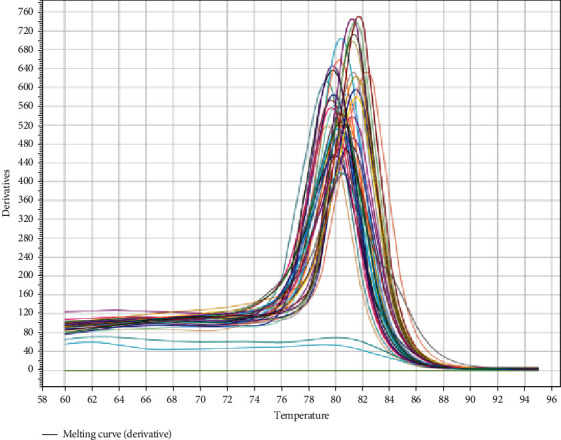
The *miR-10b* expression melting curve.

One important step in the investigation of miRNA-10b expression was the normalization process using the U6 gene. Through the process, amplification plots for the target miRNA-10b and U6 were found, which made it easier to calculate the cycle threshold (Ct) value for each gene. These Ct values and amplification plots are presumably shown in Figures 1 and 2 of the paper. These numbers are crucial in providing important information on the expression levels of miRNA-10b in the research samples, which helps evaluate the study's conclusions.

**Figure 3:**
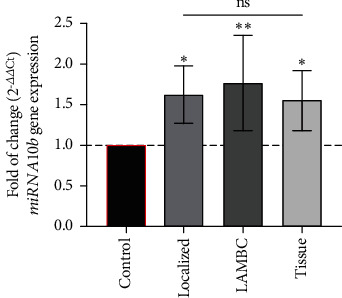
Fold of change for the gene expression of *miRNA-10b*. ^∗^LAMBC stands for locally advanced and metastatic breast cancer.

The investigation revealed a significant increase in the levels of miRNA-10b among patients with metastatic and locally advanced BC, comparing with both localized BC patients and the cohort with malignant BC tissue, specifically as follows:

- In patients with metastatic and locally advanced BC, the fold mean of miRNA-10b exhibited a significant increase, measuring 1.770 ± 0.1070 folds compared with the control group
- Among localized BC patients, the relative expression of miRNA-10b demonstrated an increase of 1.624 ± 0.064 folds
- Within the malignant BC tissue group, a significant upregulation in miRNA-10b expression was observed compared to the control group; the fold change was 1.546 ± 0.06754 as illustrated in Figure 3

These findings underscore the potential significance of elevated miRNA-10b expression, particularly in advanced stages of BC, suggesting its involvement in disease progression.

miRNAs display distinct expression patterns within cancer cells and tissues, and their detectability in bodily fluids, along with relatively high chemical stability, positions them as promising candidates for noninvasive tumor markers. In alignment with this, the upregulation of miR-10b expression was observed across disease's different stages. Specifically, in patients of localized BC, the fold change in miR-10b expression was as follows: in stage 0 (1.63), stage I (1.62), and stage IIA (1.58), as indicated in Table 2. This underscores the potential value of miR-10b as a biomarker for breast cancer, particularly in the context of disease staging.

In our study, a significant increase in the serum levels of miR-10b was observed in breast cancer patients compared to healthy controls, revealing a noteworthy average fold change. Elevated miR-10b levels harbor the potential to enhance the migratory and invasive capabilities of various cancer cells, thereby facilitating metastasis. This effect is achieved through the targeting of multiple tumor suppressor genes or metastasis suppressor genes, including notable candidates such as HOXD10, NF1, KLF4, and PTEN. Among metastasis-associated miRNAs, miR-10b, as identified by Ma et al. in 2007 [18], stands out for its involvement in regulating multiple functional targets associated with metastasis. Numerous studies corroborate these findings, demonstrating that overexpression of miR-10b can indeed augment the invasive and metastatic capabilities of cancer cells. Moreover, a substantial upregulation in the expression level of miRNA-10b was observed in breast cancer patients, with a progressive increase noted in the advanced stages of the disease. Importantly, the expression of miRNA-10b was significantly higher in metastatic breast cancer cases compared to nonmetastatic ones, consistent with findings from prior studies [19–21].

Ibrahim et al. [22] also reported the upregulation of miR-10b in a cohort of BC patients, a result consistent with the findings from the recent study conducted by Ali et al. in 2022 [23]. Aligning with these observations, Ali et al. found that serum miR-10b levels were upregulated in BC patients compared to healthy controls. Another study presented by Dwedar et al. revealed that the expression level of circulating miR-10b in BC patients was elevated when compared to controls. Furthermore, miR-10b was identified as being upregulated in metastatic BC cells and implicated in tumor invasion and metastasis [24].

Patients with metastatic or locally advanced BC had their miR-10b expression upregulated, according to the research. More specifically, the following were the fold changes in miR-10b expression during distinct phases of the disease: according to Table 3, the following increases were observed: 1.65-fold in stage IIB, 1.93-fold in stage III, 1.62-fold in stage IV recurrence, and 1.36-fold in stage IV de novo. A negative correlation between miR-10b expression levels and BC malignancy grade is demonstrated by these results. This miR-10b has the ability to be a useful biomarker for differentiating between stages of the illness, as there is a considerable variation in expression levels between stages I and III.

There was an increase in miR-10b expression in breast cancer cells, according to the research. More specifically, the following fold variations in miR-10b expression were observed for different degrees of malignancy: as seen in Table 4, there was a 1.57-fold rise in grade I, a 1.60-fold increase in grade II, and a 1.47-fold increase in grade III. The results highlight the increased expression of miR-10b in breast cancer and suggest a correlation between the degree of malignancy and this gene, with the largest fold increase seen in grade II cancer. Understanding the aggressiveness and course of cancer may be possible through the differential expression of miR-10b across distinct malignancy grades.

There is a lot of hope that miR-10b, a prometastatic microRNA, might be a therapeutic target for cancer. As a potential target for antimetastatic medicines, it also acts as a powerful diagnostic tool for BC therapy [25]. The miR-10b pathway is essential for the invasion, colonization, and metastasis of BC cells to the lungs [26, 27]. The evaluation of miR-10b highlights its possible diagnostic use, sparking conversations on its function as a serum marker in the diagnosis and monitoring of BC therapy. Specifically, in high-grade human BC, miR-10b is more highly expressed and promotes VEFG synthesis, according to new studies published in 2021 [28] by Yoo et al. [29]. In addition, it should be noted that miR-10b is secreted by metastatic BC cells in the form of exosomes. When nonmalignant mammary epithelial cells absorb this miR-10b, it causes them to become invasive [30]. Together, our results demonstrate that miR-10b is essential for the development and spread of BC, and they draw attention to the fact that it may be useful in diagnostic and therapeutic settings.

## 4. Conclusion

miR-10b emerges as a key driver in promoting tumor progression and metastasis across various cancer types. Initial observations revealed elevated levels of miR-10b not only in metastatic tumors but also in primary tumors from individuals with metastatic BC. Further investigations have unveiled that high miR-10b expression is closely linked with high-grade malignancy and metastasis in BC. Notably, lymph node metastases exhibit higher levels of miR-10b compared to corresponding primary tumors in multiple types of human cancer. The pivotal role of miR-10b lies in its ability to advance the invasion and migration of tumor cells, making it a crucial driver of metastatic cell survival and enabling their growth outside the primary tumor site. These outcomes have spurred the development of strategies aimed at treating metastatic cancer by inhibiting miR-10b.

Recent studies have illuminated the fact that BC cells can secrete miR-10b via exosomes, thereby promoting tumor progression and development. The multifaceted roles of miR-10b in initiating and advancing tumor metastasis underscore its significance as a potential therapeutic target in cancer. Ongoing research focusing on therapeutics involving miR-10b inhibition holds the potential to enhance BC management and decrease cancer-related mortality rates. The expression of miRNA-10b gene exhibited a significant increase in Iraqi women patients with breast cancer, particularly in metastatic stages compared to other stages.

## Figures and Tables

**Table 1:** Thermal profile of *miRNA-10b* gene expression.

Cycle step	Temperature	Time	Cycles
Initial denaturation	95°C	8 min	1
Denaturation	95°C	15 sec	50
Extension	60°C	30 seconds (+plate read)	
Melt curve	60-95°C	40 min	1

**Table 2:** Fold change in miR-10b expression among patients with localized BC group, stratified by disease stages.

Genes	Folding		
	Stage 0	Stage 1	Stage 2A
*miR-10b*	1.63a	1.62a	1.58a
LSD at 0.05 probability			

**Table 3:** Fold change in miR-10b expression among patients with locally advanced and metastatic breast cancer group, stratified by disease stages.

Genes	Folding			
	Stage III	Stage IIB	Stage IV recurrence	Stage IV de novo
*miR-10b*	1.93a	1.65a	1.62a	1.36a
LSD at 0.05 probability				

**Table 4:** Fold change in miR-10b expression in malignant breast cancer tissue among women, stratified by disease grade.

Genes	Folding		
	Grade I	Grade II	Grade III
*miR-10b*	1.57a,b	1.60b	1.47a
LSD at 0.05 probability			

## Data Availability

Data are provided as requested.

## Supplementary Materials

Supplementary Materials Table S1: reaction volume and contents of reverse transcription reaction utilized to prepare cDNA from total RNA. Table S2: component of qRT-PCR used in *miRNA-10b* and *U6* expression experiment.
