# Supplementary material for: Assessment of miRNA-10b Expression Levels as a Potential Precursor to Metastasis in Localized and Locally Advanced/Metastatic Breast Cancer among Iraqi Patients
Source: Int J Breast Cancer. 2024 Feb 28;2024:2408355. doi: 10.1155/2024/2408355 (PMC10917482; doi:10.1155/2024/2408355)
Supplement: Supplementary Materials — Table S1: reaction volume and contents of reverse transcription reaction utilized to prepare cDNA from total RNA. Table S2: component of qRT-PCR used in miRNA-10b and U6 expression experiment. [file 2408355.f1.docx]

**Supplementary Information**

**Assessment of miRNA-10b Expression Levels as a Potential Precursor to Metastasis in Localized and Locally Advanced/Metastatic Breast Cancer Among Iraqi Patients**

Mays Talib Abdullah^1,*^, Ismail Aziz^1^, Ahmed Zuhair Alsammarraie^2^,

^1^ Institute of Genetic Engineering and Biotechnology, University of Baghdad, Baghdad, Iraq

^2^ Oncology Teaching Hospital, Baghdad Medical City, Baghdad, Iraq

^3^ Department of Environmental Science, College of Energy and Environmental Science, Alkarkh University of Science, Baghdad, 10081, Iraq.

* Corresponding Author Email: Mays Abdullah: <mailto:mais.taleb1100a@ige.uobaghdad.edu.iq>

**Table S1. Reaction volume and contents of reverse transcription reaction utilized to prepare cDNA from total RNA**.

| **Contents** | **Volume (µl) Reaction** |
| --- | --- |
| TransScript® miRNA RT Enzyme Mix | 1µl |
| Total RNA | 6 µl |
| 2×TS miRNA Reaction Mix | 10 µl |
| RNase-free water | To 20 µl |

**Table S2. Component of qRT- PCR used in *miRNA10b*, *U6* expression experiment**.

| **Components** | **Volume (**$\boldsymbol{\mu l}$**)** | **Concentration** |
| --- | --- | --- |
| Master mix syper green | 10 |  |
| Forward primer | 0.5 | 10 pmol |
| Reverse primer | 0.5 | 10 pmol |
| CDNA | 6 |  |
| Nuclease-free water (N.F.W) | 3 |  |
| Total | 20 |  |
